# Tackling Diversity in Prostate Cancer Clinical Trials: A Report From the Diversity Working Group of the IRONMAN Registry

**DOI:** 10.1200/GO.20.00571

**Published:** 2021-04-09

**Authors:** Rana R. McKay, Theresa Gold, Jelani C. Zarif, Ilkania M. Chowdhury-Paulino, Adam Friedant, Travis Gerke, Marie Grant, Kelly Hawthorne, Elisabeth Heath, Franklin W. Huang, Maria D. Jackson, Brandon Mahal, Osarenren Ogbeide, Kellie Paich, Camille Ragin, Emily M. Rencsok, Stacey Simmons, Clayton Yates, Jake Vinson, Philip W. Kantoff, Daniel J. George, Lorelei A. Mucci

**Affiliations:** ^1^ Moores Cancer Center, University of California San Diego, La Jolla, CA; ^2^ Prostate Cancer Clinical Trials Consortium, New York, NY; ^3^ Johns Hopkins University School of Medicine, Baltimore, MD; ^4^ Harvard University, Boston, MA; ^5^ Moffitt Cancer Center, Tampa, FL; ^6^ Movember Foundation, East Melbourne, Victoria, Australia; ^7^ Karmanos Cancer Center, Detroit, MI; ^8^ University of California San Francisco, San Francisco, CA; ^9^ University of the West Indies, Mona, Kingston, Jamaica, West Indies; ^10^ University of Miami, Miami, FL; ^11^ University of Tennessee, Memphis, TN; ^12^ Fox Chase Cancer Center, Philadelphia, PA; ^13^ African-Caribbean Cancer Consortium, Philadelphia, PA; ^14^ Bayer Healthcare Pharmaceutical, Wayne, NJ; ^15^ Tuskegee University, Tuskegee, AL; ^16^ Prostate Cancer Transatlantic Consortium (CaPTC), Jacksonville, FL; ^17^ Memorial Sloan Kettering Cancer Center, New York, NY; ^18^ Duke University, Durham, NC

## Abstract

Prostate cancer disproportionately affects racial and ethnic minority populations. Reasons for disparate outcomes among minority patients are multifaceted and complex, involving factors at the patient, provider, and system levels. Although advancements in our understanding of disease biology have led to novel therapeutics for men with advanced prostate cancer, including the introduction of biomarker-driven therapeutics, pivotal translational studies and clinical trials are underrepresented by minority populations. Despite attempts to bridge the disparities gap, there remains an unmet need to expand minority engagement and participation in clinical trials to better define the impact of therapy on efficacy outcomes, quality of life, and role of biomarkers in diverse patient populations. The IRONMAN registry (ClinicalTrials.gov identifier: NCT03151629), a global, prospective, population-based study, was borne from this unmet medical need to address persistent gaps in our knowledge of advanced prostate cancer. Through integrated collection of clinical outcomes, patient-reported outcomes, epidemiologic data, and biospecimens, IRONMAN has the goal of expanding our understanding of how and why prostate cancer outcomes differ by race and ethnicity. To this end, the Diversity Working Group of the IRONMAN registry has developed informed strategies for site selection, recruitment, engagement and retention, and trial design and eligibility criteria to ensure broad inclusion and needs awareness of minority participants. In concert with systematic strategies to tackle the complex levels of disparate care, our ultimate goal is to expand minority engagement in clinical research and bridge the disparities gap in prostate cancer care.

## INTRODUCTION

Over the past 15 years, the treatment landscape for advanced prostate cancer has undergone tremendous change. Given an improved understanding of disease biology and the development of rationally designed therapies, we have witnessed an expansion of treatments for patients. Despite an increasing number of therapies for patients with castration resistant prostate cancer and earlier use of treatments for hormone-sensitive prostate cancer, many gaps exist in our understanding of utilization in the real-world including patient selection of treatment, optimal treatment sequences and combinations, and toxicity. Studies capturing the patient experience and clinical decision making regarding physician choice of therapy are still lacking.

**CONTEXT**

**Key Objective**
How does the IRONMAN registry overcome barriers to diversity involvement in prostate cancer clinical trials? The IRONMAN study was launched to fill the knowledge gaps and expand our understanding of the impact of race and ethnicity on a broad spectrum of prostate cancer outcomes.
**Knowledge Generated**
The Diversity Working Group of the IRONMAN registry has developed informed strategies for trial design, eligibility, site selection, site and participant recruitment, and retention to ensure the broad inclusion and needs awareness of minority participants. We highlight these strategies and review the lessons learned to expand minority representation in prostate cancer clinical trials.
**Relevance**
The IRONMAN registry can serve as a model for disparity-focused research. Systematic efforts will be necessary to revamp the existing clinical trials construct to promote representation of minority groups in clinical research.

In the United States, Black men have an increased incidence of prostate cancer and worse mortality.^1^ Among Hispanic individuals, Puerto Rican men also experience an increased incidence and worse prostate cancer mortality compared with non-Hispanic White men.^2^ Existing prostate cancer clinical trials do not accurately reflect the diverse populations of men suffering from this disease nor those who are high-risk.^3^ Although Black men represent 13.4% of the US population, they represent 6.7% of patients enrolled on therapeutic prostate cancer clinical trials.^3^ The disparity is even greater for those of Hispanic or Latino origin. This group represents 19% of the US population, but < 2% of prostate cancer therapeutic clinical trial participants.^3^ Disparities in clinical trial enrollment further exacerbate disparities in outcomes among men of diverse racial and ethnic groups. The underrepresentation of minorities in clinical research blunts our ability to resolve these disparities gaps and improve outcomes for minority patients. Additionally, the accelerating focus on precision medicine and unique tumor profiles, as a function of race and ethnicity, has heightened the urgency to bridge inequities in clinical research.

IRONMAN is a global registry of men with advanced prostate cancer (ClinicalTrials.gov identifier: NCT03151629). This prospective study aims to bridge the gap in information obtained from randomized clinical trials to real-world clinical practice. With the goal of translating this new knowledge to tangible benefits for patients, IRONMAN focuses on capturing clinical outcomes, patient-reported outcomes, epidemiologic data, and clinical decision making in men with advanced prostate cancer with focus on minority patients. Furthermore, through longitudinal specimen collection, IRONMAN is positioned to evaluate biomarkers of response and resistance across racial and ethnic groups. The Diversity Working Group was established in 2017 to maximize racial and ethnic diversity in the IRONMAN study population and ensure prioritization of initiatives focused on improving outcomes for minority patients. Despite the plethora of studies describing the disparate outcomes in prostate cancer, few describe strategies to overcome these hurdles in clinical research. Herein, we intend to critically appraise challenges to prostate cancer clinical trial enrollment. We offer potential solutions using published work and our first-hand experience engaging in disparity research, which has been incorporated in the IRONMAN registry. Our ultimate goal is to stimulate a robust knowledge exchange on how to improve minority access to clinical trials with the hope of eliminating prostate cancer health disparities.

## OBSERVATIONAL STUDIES IN PROSTATE CANCER RESEARCH

High-quality observational studies play a fundamental role in providing information to improve medical decision making for men with advanced prostate cancer. Although randomized controlled trials have a clear place in establishing efficacy, gaps remain in our medical knowledge regarding patient selection, treatment sequences, and toxicity management in real-world clinical practice. Observational studies can provide valuable information that is complementary to evidence derived from randomized controlled trials. Observational research studies have been instrumental in identifying differences in health outcomes based on race and ethnicity and improving our knowledge of the factors that contribute to these disparities. Given feasibility and generalizability challenges associated with randomized clinical trials, observational studies have become the mainstay of health disparities research.^4^

## DEFINING RACE, ETHNICITY, AND ANCESTRY

There is significant complexity in defining racial and ethnic minority groups. In general, race classifications have emphasized geographic region of origin of a person's ancestry.^5^ Ethnicity is a broader construct that accounts for cultural tradition, common history, religion, and other factors.^5^ Despite complexities in defining race and ethnic groups, capturing information in the context of clinical research is important to identifying disparities among diverse populations.

Currently, international standards for defining race and ethnicity are lacking.^6^ The existing federal classification by race and ethnicity was established by the US Office of Management and Budget in 1977 and was updated in 1997 to include the following categories: American Indian or Alaska Native, Asian, Black or African American, Native Hawaiian or Other Pacific Islander, and/or White. The two categories for ethnicity include (1) Hispanic or Latino, or (2) not Hispanic or Latino.^7^ These race and ethnicity groups are sociopolitical constructs.^8^ They are not intended to define scientific or anthropologic classifications and do not encompass the diverse range of peoples.^9^ Classification has been largely dependent on self-identification. Additionally, these categories may not be appropriate across other countries or regions; however, a basic taxonomy of racial and ethnic classifications that suggests a greater commonality worldwide is lacking.

Improvements in capabilities to perform large-scale genomic investigations have provided an opportunity to determine genetic ancestry.^10^ In a diverse admixed population, limitations may exist with using self-reported race and ethnic categories as proxies to genetic ancestry.^11^ Ancestry-based health disparity studies provide a new way to dissect the contribution of genetics to health disparities from nongenetic factors.^10^

## DISPARITIES IN PROSTATE CANCER OUTCOMES

Although prostate cancer is the most common cancer among men and one of the leading causes of death worldwide, certain groups bear a disproportionate burden of disease.^12,13^ In the United States, incidence and mortality rates (per 100,000) are highest among Black men (173.0 and 38.7, respectively) in comparison with White men (97.1 and 18.0, respectively), American Indian or Alaska natives (68.0 and 18.7, respectively), and Asian or Pacific Islander (52.9 and 8.6, respectively).^1,14,15^ Aggressive prostate cancers are relatively more common in Black men.^1,16,17^ Among Hispanic men, prostate cancer is the most diagnosed cancer (21%); however, incidence and mortality are lower compared with non-Hispanic White men (incidence 91.6 *v* 101.7; mortality 15.9 *v* 18.1).^2^ Among men in Puerto Rico, the incidence is 44% higher relative to non-Hispanic White men and prostate cancer is the leading cause of cancer death in men.^2^

Significant differences in prostate cancer mortality are also dependent on birthplace. Immigrant Black men from Africa and the Caribbean have lower prostate cancer mortality in comparison with US-born Black men.^18^ Among immigrant Hispanics, prostate cancer mortality varies, where Dominican men have the highest mortality rates and Mexican men have the lowest.^14,18^ A regional study in California found that foreign-born Hispanic men were more likely to be diagnosed with advanced stage and grade disease when compared with US-born Hispanic men.^19^ Similar patterns were observed when evaluating US-born and Caribbean-born Black men.^18^ These studies highlight that nativity and ancestral factors are likely critical components in understanding disparate outcomes in men with prostate cancer.

Recent global estimates demonstrate that the highest incidence rates of prostate cancer were found in Australia or New Zealand, Northern America, Western and Northern Europe, and the Caribbean. Mortality patterns differed with the highest estimated rates in the Caribbean, sub-Saharan Africa, and parts of the former Soviet Union.^12^ Although prostate cancer incidence is decreasing in the United States,^20^ low- and middle-income countries and most Asian countries have observed an increased incidence.^21^ Despite the lack of developed national cancer registries to capture all of the cancer cases in each country, Caribbean countries such as Jamaica and Martinique,^22^ and African countries such as Zimbabwe, Uganda, Nigeria, and South Africa,^23–25^ all report increases in prostate cancer incidence, highlighting the global impact of this disease.

## REASONS FOR DISPARATE OUTCOMES ON MEN WITH PROSTATE CANCER

Determining the impact of race and ethnicity on outcomes is complex. There is likely an interplay between multiple factors including racism, genetic and biologic determinants, differences in diet, physical activity, and other exposures, psychosocial factors, socioeconomic factors, and healthcare access.^26^ In 2003, the Institute of Medicine published a comprehensive report focusing on racial and ethnic disparities in health care.^27^ Key findings indicate that access to appropriate health care is dependent on health insurance coverage, income, and education. They recognized that social inequities, including the legacy of racial discrimination in the United States, can influence interactions between patients and clinicians. Additionally, cultural factors were emphasized as they can play a role in health behaviors, attitudes toward illness, and belief in modern medicine. Developing strategies to mitigate risk associated with fixed risk factors (ie, early screening for men with a genetic predisposition) or modify adaptable risk factors (ie, equitable access to care for all men) will be critical to improving outcomes for patients.

## DISPARITIES IN PROSTATE CANCER CLINICAL TRIALS

Despite these known racial disparities in prostate cancer outcomes, African Americans and other underserved ethnic and minority groups in the United States have historically been underrepresented in prostate cancer clinical trials. Less than 5% of adult cancer patients participate in a clinical trial and this disparity is even starker for minority patients.^28^ Appropriate diversity in clinical trials is critical as reduced efficacy or increased toxicity may occur when agents are brought to market after testing in nonrepresentative clinical trial populations.^29^ Regardless of enrollment trends, recent literature emphasizes underenrollment of all US minority groups.^3,30,31^

The reasons for underenrollment into clinical trials are multifaceted. This current dilemma may be rooted in a generational mistrust for health care–providing entities that has long existed in the United States. From the patient perspective, potential barriers include lack of social support, culturally inappropriate care, and minimal information about open trials and the trial process in general.^32^ Restrictive eligibility criteria, complex trial designs, and need for additional tests and visits further negatively affect minority accrual. Such processes may require additional resources, including time and cost. From the perspectives of providers, unconscious physician bias fueled by misperceptions about minority engagement may underlie barriers to enrollment and communication with minority patients. Additionally, a lack of resources to facilitate minority recruitment, limitations of translator services and clinical time to ensure informed discussions, and varied clinical trial referral patterns are likely additional contributors.^3,33^

It is important to also recognize systemic and institutional barriers to minority enrollment. Such barriers include limited availability of resources to operationalize clinical trial conduct in health practices where minority patients receive cancer care. Additionally, if trials are available, the breadth of trial options may be limited and may not be available for a substantial number of patients.^34^ A lack of community engagement and outreach to educate patients and community partners in an open and honest manner likely further exacerbates enrollment disparities. Inadequate or lack of healthcare insurance is another barrier.

The rise of precision medicine has an unintended risk of exacerbating preexisting health disparities.^35,36^ In addition to participation in biomarker-driven precision medicine clinical trials, access to next-generation sequencing and participation in biospecimen repositories are areas that require focus. Although next-generation sequencing provides information that can lead to personalized treatment, it requires availability of tissue or blood specimens, a payer willing to reimburse for the test, and a provider willing to order the test. Each of these steps presents the potential hurdle that disproportionately affects minorities. Although large-scale efforts gathering genomic information have emerged, such as The Cancer Genome Atlas, many underrepresent minority populations.^37^ The inclusion of underrepresented populations in these repositories and development of warm autopsy programs will provide relevant genomic insights pertinent to specific groups of patients.

## CALL TO ACTION TO BRIDGE THE DISPARITIES GAP IN CLINICAL TRIALS

The US population is growing and by 2045, nearly half of the population is expected to be other than non-Hispanic White individuals. This mandates a need for proactive planning, corrective measures, and systematic interventions to improve disparate outcomes for men with prostate cancer.^38^ Without such deliberate and effective interventions, the disparities chiasm will likely widen in the decades to come. The National Institutes of Health Revitalization Act of 1993 mandated the inclusion of women and racial or ethnic minorities into National Institutes of Health–supported clinical research trials.^39^ Although the ultimate goal was to ensure that all people fully benefit from advances in biomedical research, this federal mandate failed to notably increase minority enrollment.^40,41^ Subsequently, Section 907 of the Food and Drug Administration Safety and Innovation Act of 2012 was implemented requiring reporting of demographic subgroups in trials and subsequent action plan in 2015.^42^ In 2019, the Food and Drug Administration issued a draft guidance recommending approaches that sponsors of clinical trials can take to broaden eligibility criteria and increase enrollment of underrepresented populations.^43^

In addition to initiatives that target sponsor practices, there is a call to reform multiple aspects of care delivery and clinical trial conduct to bridge the disparities gap in prostate cancer clinical trials. This includes a focus on patient, clinician, institution, and health systems strategies to resolve systematic inequities in clinical trials. Engagement of stakeholders across these levels will be critical to effect meaningful changes in clinical trial operations.

## THE IRONMAN REGISTRY

### History of Development

The IRONMAN registry was launched in 2016 to address the unmet needs among men with advanced prostate cancer. At the time, the clinical landscape of advanced prostate cancer was (and is still) rapidly changing. As a consequence, there lacked consensus on the optimal sequences or combination of these new therapies for men with advanced prostate cancer. Coupled with this was an understanding that biomarkers held promise to guide the optimal treatments for men with emerging evidence of the variability in clinical outcomes across biologic subsets of the disease. A major gap the project sought to address was the importance of patient-reported outcomes as a pivotal component of understanding quality of life and treatment benefits for patients with cancer, yet comprehensive assessment in advanced prostate cancer was lacking. Finally, given the marked disparities in prostate cancer outcomes, the registry sought to understand and bridge these gaps within different populations of men. One unique feature of this registry and its development has been the partnerships across diverse stakeholders: clinicians, public health practitioners, the pharmaceutical industry, advocacy, and a men's health foundation. Although the scope of the IRONMAN registry is focused at capturing the multifaceted clinical and therapeutic outcomes of men with advanced prostate cancer, one of the multiple key priorities is understanding the impact of race and ethnicity on factors that affect advanced prostate cancer care and outcomes.

### Study Design

The IRONMAN registry is a global prospective, population-based study of men with advanced prostate cancer, including men with newly diagnosed metastatic hormone-sensitive prostate cancer and metastatic or nonmetastatic castration-resistant prostate cancer. The goal is to recruit a minimum of 5,000 patients and include men from sites around the world. By recruiting men from different populations, we intend to leverage differences across the populations to better understand and improve outcomes for men with advanced prostate cancer. Institutional review board approval and participant informed consent is required.

**FIG 1:**
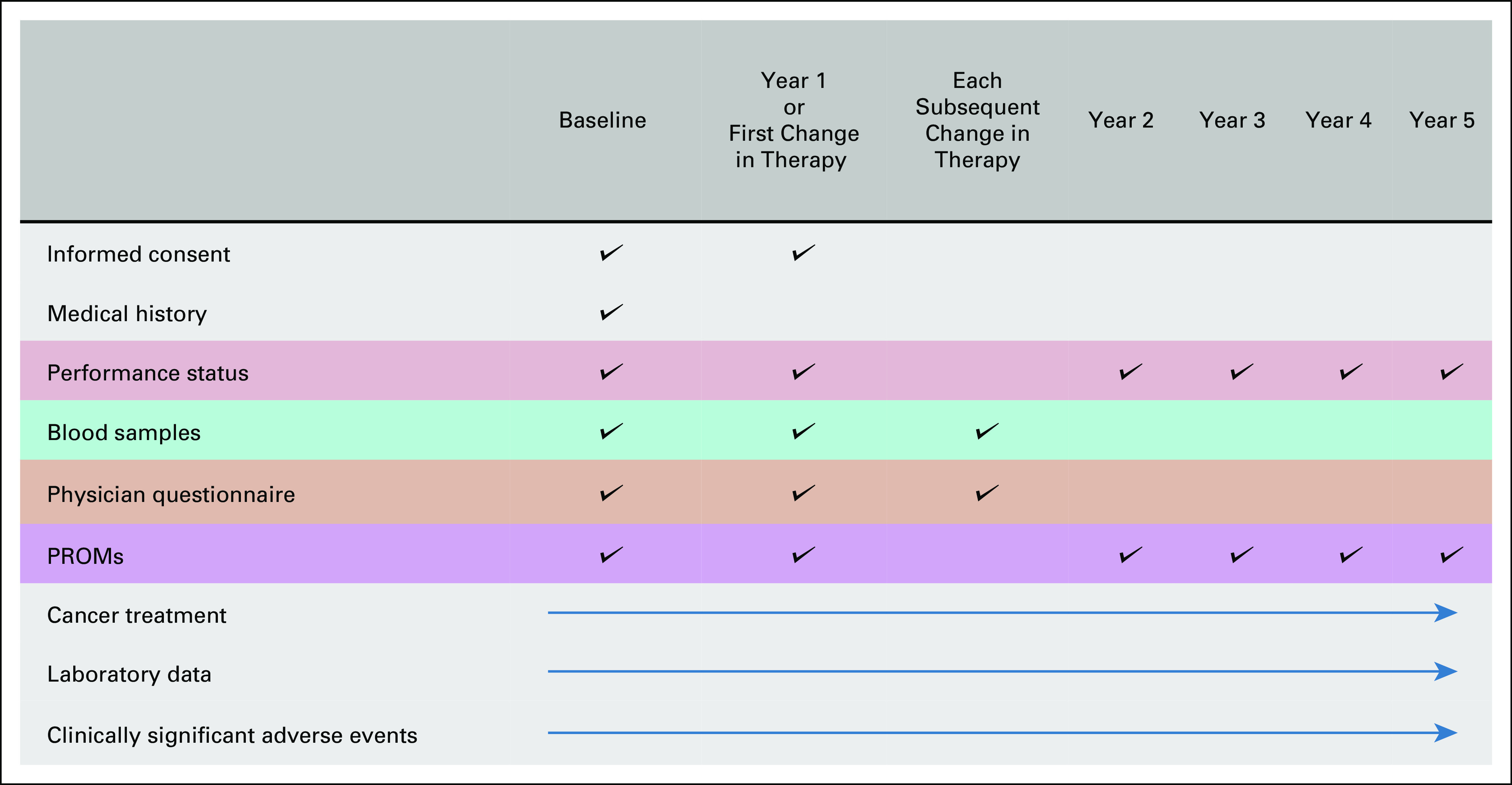
IRONMAN study procedures. PROMs, patient-reported outcome measures.

Detailed data will be collected at study enrollment and during follow-up, for a minimum of five years (Fig 1). Patients will be followed prospectively for overall survival, clinically significant adverse events, comorbidities, changes in cancer treatments, and patient-reported outcomes measures (PROMs). PROMs questionnaires will be collected at enrollment and every three months and cover domains of pain, fatigue, mental health, sexual health, urinary health, cognitive function, and sleep quality. Additionally, the PROMs capture the social and financial impacts of treatment. Physician Questionnaires will be collected from all participating sites at enrollment, time of first change in treatment, at each subsequent change of treatment, and discontinuation of treatment. The Physician Questionnaire captures reasons for treatment discontinuation including financial cost or change in insurance status.

Blood samples will be collected at enrollment, time of first treatment change because of progression of disease or at the 12-month follow-up visit if stable, and at each subsequent change in treatment because of progression of disease. Current efforts have focused on ensuring broad and timely collection of specimens from all study participants before embarking on specific biomarker studies.

In the IRONMAN registry, race and ethnicity will be captured from self-reporting. Understanding that applicability of predefined race and ethnic categories across multiple global regions may be limited, collection of blood from all participants will allow for coupling self-reported race with genetically determined ancestry for minority subpopulation evaluation. Other social determinants of health that will be captured include education level, employment status, and regional socioeconomic status.

### Diversity Working Group

The working groups of the IRONMAN registry were assembled to address the primary needs and initiatives of the study. They include the Low and Middle Income Countries, Advocacy, PROMs, Physician Questionnaire, Biospecimen, Clinical Research Coordinator, and Statistics Working Groups. In 2017, members of the IRONMAN executive committee invited a team of prostate cancer experts to form the Diversity Working Group. The primary goal of the working group from its inception has been to recruit a diverse patient population into the IRONMAN registry (Table 1). The working group appreciated that diversity was multifaceted and included representation from both rural and urban settings, cancer centers and local group practices, groups of differing socioeconomic status, and patients of diverse ancestry. Although diversity is broadly defined, the Diversity Working Group prioritized minority enrollment with a focus on men of African descent. The group defined tangible accrual goals to measure success. To ensure capture of minority patients, the Diversity Working Group developed a strategic outreach plan to target active sites in communities with an increased population of minority patients. As part of this outreach plan, the Diversity Working Group queried site investigators and clinical trials staff to understand barriers to minority enrollment and developed strategies unique to the IRONMAN registry to expand enrollment.

**TABLE 1:**
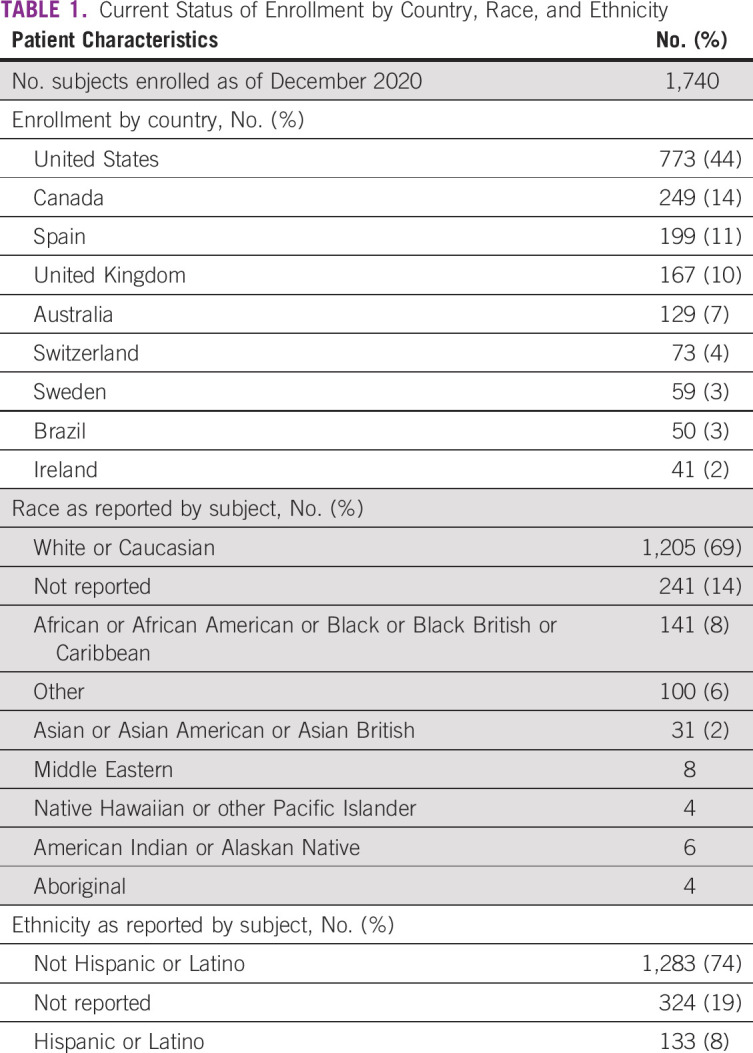
Current Status of Enrollment by Country, Race, and Ethnicity

### Strategies to Enhance Minority Engagement

Strategies to enhance minority representation and broaden the scope of the IRONMAN registry have permeated all aspects of the study including study design, site selection, study team support, and international collaborations (Table 2). When IRONMAN was initially launched, the number of sites was limited in scope. Given the overarching mission of the study, there was a critical need to engage with nontraditional sites to ensure inclusivity and appropriate representations of a broad scope of men with prostate cancer. Partnerships with the African-Caribbean Cancer Consortium and the Prostate Cancer Transatlantic Consortium were developed with the goal to open the study in the Bahamas, Jamaica, Barbados, Nigeria, and Kenya. The African-Caribbean Cancer Consortium was formed in 2006 to further the study of viral, genetic, environmental, and lifestyle risk factors for cancer in populations of African descent. The consortium consists of three interconnected regional networks (United States, Africa, and the Caribbean) of multidisciplinary teams including cancer advocates, basic, translational, and population science researchers, and clinicians.^44^ The Prostate Cancer Transatlantic Consortium was formed in 2005 to address the globally disproportionate burden of prostate cancer among Black men. It is an open consortium comprising a team of prostate cancer scientists, clinicians, survivors, and advocates from North America, Europe, the Caribbean Islands, and West Africa. Additionally, given the high prevalence of prostate cancer among US veterans and relatively larger proportion of minority individuals receiving care in this Veterans Affairs healthcare system, efforts are underway to facilitate participation with US Veterans Affairs sites. Furthermore, the Low and Middle Income Countries Working Group was launched in 2019 with the goal of expanding site selection to less resourced global regions. The current accrual numbers do not reflect these efforts as sites are currently in the midst of activation. Additional efforts to enhance minority recruitment have focused on partnerships with advocacy groups to ensure awareness and promotion of the initiative within the broader community and patient networks. Both the Diversity Working Group and Executive Committee continually review the state of enrollment in IRONMAN to ensure benchmarks of success. Between July 2017 and December 2020, 1,740 men have been enrolled (Table 2).

**TABLE 2:**
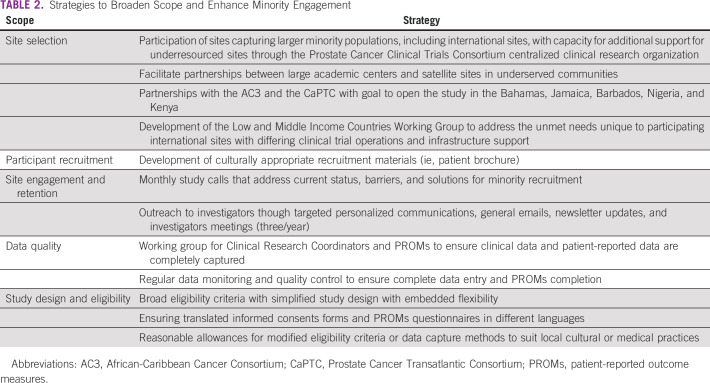
Strategies to Broaden Scope and Enhance Minority Engagement

### Lessons Learned

**FIG 2:**
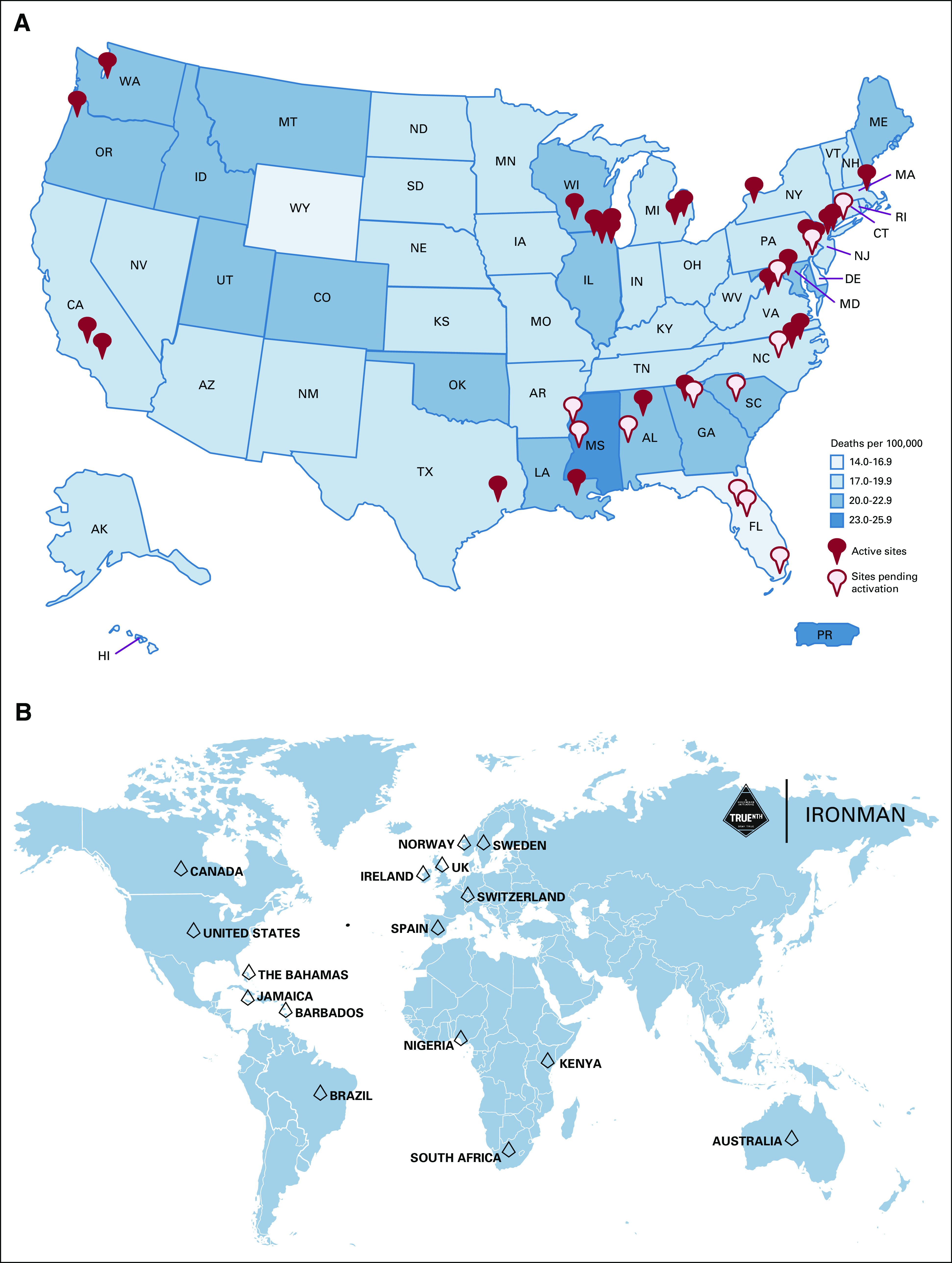
(A) Active and pending IRONMAN sites superimposed over map of prostate cancer mortality by state. (B) Countries with participating international sites worldwide.

As the IRONMAN registry continues to expand in enrollment and geography, we have learned from our initial experience in onboarding the first 35 US and 55 international sites (Fig 2). We learned early on that it was critical to outreach and involve sites that predominately serve underrepresented populations to achieve equity in representation of not just individuals but nontraditional practices and hospital systems. One of the key principals for success in collaborating with investigators, health systems, and communities of variable research infrastructure has been the identification of a study champion at each site committed to the mission of IRONMAN. The lead investigators at each of the sites of interest had a great understanding of their local community needs, available resources, and were able to assemble the study team to facilitate the activation of the study and patient enrollment.

In collaborating with many international sites, we learned that defining diversity was variable across the globe. Empowerment of country leadership was important to help shape the diversity goals and accrual targets for each site. This was particularly relevant as we developed partnerships in Brazil, the Caribbean, and parts of Africa.

We learned throughout the process that communication and adaptability were critical to ensuring our overarching goals were achieved. A continual feedback mechanism with sites, investigators, and patient advocates through structured meetings and forums was critical to understanding the unique needs of each group. This open communication process allowed for the identification of barriers and joint development of solutions to overcome challenges. This was particularly important in defining eligibility windows and enrollment criteria to ensure broad participation, implementation of the PROMs and physician questionnaires to ensure completion and data capture, and collection of biospecimens. Although these issues are not unique to minority subpopulations, the Diversity Working Group ensured that they did not disproportionally affect minority participation.

### Impact of the COVID-19 Pandemic

**FIG 3:**
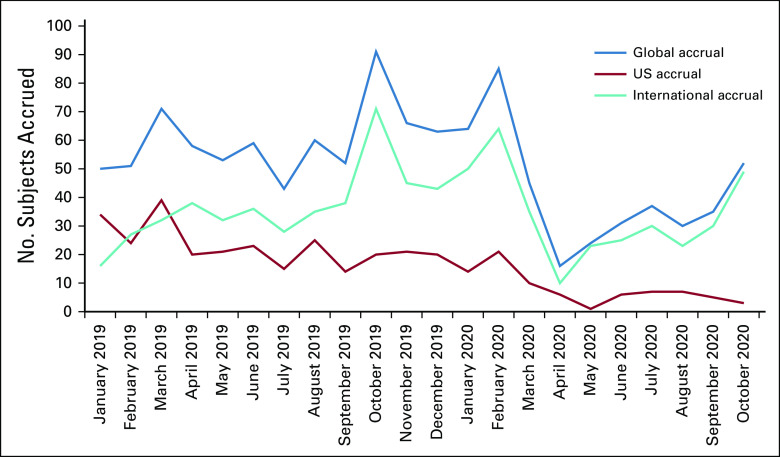
Monthly IRONMAN study accrual. Blue, global study wide accrual; teal, international (non-US site) accrual; red, US accrual.

The COVID-19 pandemic has affected clinical trials including IRONMAN, and the study team has adapted to ensure the safety of its participants, clinicians, and research staff (Table 3). Given geographic differences in COVID-19 prevalence, severity, and variability in regional and institutional public health policies, the degree of impact on enrollment and site activation has been variable. Given the nontherapeutic nature of the study, many institutions halted enrollment during the peak of the pandemic. Although enrollment in the United States declined during this time, international enrollment increased (Fig 3). The silver lining has been the opportunity to modify protocol-specific procedures for adherence and retention of participants without compromising the study analytic plan. The introduction of telehealth and remote trial procedures (remote consenting, monitoring, and electronic PROMs) has also enabled continuation of study activities. Given challenges with biospecimen collection, potential solutions including home phlebotomy and dried blood spot collection are currently being developed to facilitate continued specimen collection. Furthermore, we instituted a physician questionnaire to capture the impact of COVID-19 on care patterns of IRONMAN participants at the local level.

**TABLE 3:**
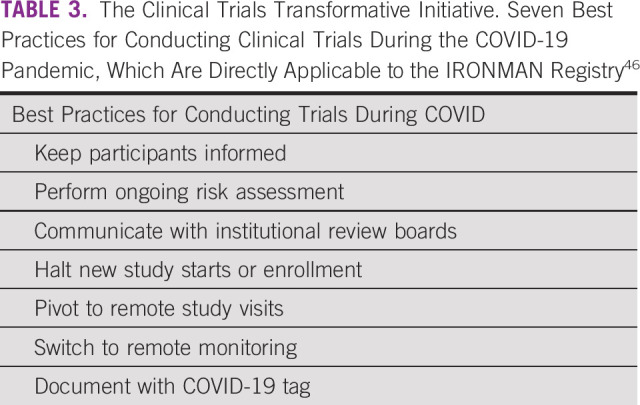
The Clinical Trials Transformative Initiative. Seven Best Practices for Conducting Clinical Trials During the COVID-19 Pandemic, Which Are Directly Applicable to the IRONMAN Registry^46^

Patients with prostate cancer share risk factors for COVID-19 disease severity: male, older age, metastatic disease, and minority populations. At the same time, men with advanced prostate cancer are on active treatment. The Diversity Working Group thus developed a physician questionnaire to capture alterations in care and modifications in treatment plans for patients enrolled on the IRONMAN registry. Additionally, clinically significant adverse events as a result of COVID-19 as well as COVID-19 mortality are being captured on the electronic case report forms. The ultimate goal of these study adaptations is to ensure the continued protection of the participants enrolled on the study, while mitigating risks to the integrity of the trial.^45^

In conclusion, racial and ethnic minority populations experience a higher burden of prostate cancer but have been underrepresented in clinical trials. Although the treatment landscape for advanced prostate cancer has been rapidly evolving with new treatments, there is limited understanding of the impact of race and ethnicity on the efficacy of approved agents, given the low number of minority patients enrolled on pivotal trials. Additionally, although our knowledge of genomic drivers of disease has expanded and resulted in the introduction of molecularly targeted therapies for prostate cancer, underrepresentation of minority patients in these translational and clinical studies has limited application to minority patients. Understanding the need to proactively and deliberately address these challenges in current research, the IRONMAN study was launched to fill the knowledge gaps and expand our understanding of the impact of race and ethnicity on a broad spectrum of prostate cancer outcomes. Although the IRONMAN study is a model for disparity focused research, additional systematic efforts are needed to revamp the existing clinical trials construct to promote representation of minority groups in clinical research.

## Data Availability

A data sharing statement provided by the authors is available with this article at DOI https://doi.org/10.1200/GO.20.00571.
